# Prevalence and correlates of dizziness in community-dwelling older people: a cross sectional population based study

**DOI:** 10.1186/1471-2318-13-4

**Published:** 2013-01-04

**Authors:** Suzana Albuquerque de Moraes, WuberJeffersondeSouza Soares, Eduardo Ferriolli, Monica Rodrigues Perracini

**Affiliations:** 1 Physical Therapy Department, City of Sao Paulo University, Sao Paulo, Brazil; 2 Faculty of Medicine of Ribeirao Preto, University of Sao Paulo, Ribeirao Preto, Brazil; 3 Faculty of Medicine, State University of Campinas, Sao Paulo, Brazil; 4 Visiting Research Fellow at Sydney University and The George Institute for Global Health, Sydney, Australia

**Keywords:** Aged, Depression, Dizziness, Elderly adults, Fatigue, Sleep disorders by excessive drowsiness, Cross sectional study

## Abstract

**Background:**

Dizziness is a common complaint among older adults and has been linked to a wide range of health conditions, psychological and social characteristics in this population. However a profile of dizziness is still uncertain which hampers clinical decision-making. We therefore sought to explore the relationship between dizziness and a comprehensive range of demographic data, diseases, health and geriatric conditions, and geriatric syndromes in a representative sample of community-dwelling older people.

**Methods:**

This is a cross-sectional, population-based study derived from FIBRA (Network for the Study of Frailty in Brazilian Elderly Adults), with 391 elderly adults, both men and women, aged 65 years and older. Elderly participants living at home in an urban area were enrolled through a process of random cluster sampling of census regions. The outcome variable was the self-report of dizziness in the last year. Several feelings of dizziness were investigated including vertigo, spinning, light or heavy headedness, floating, fuzziness, giddiness and instability. A multivariate logistic regression analysis was conducted to estimate the adjusted odds ratios and build the probability model for dizziness.

**Results:**

The complaint of dizziness was reported by 45% of elderly adults, from which 71.6% were women (*p*=0.004). The multivariate regression analysis revealed that dizziness is associated with depressive symptoms (OR = 2.08; 95% CI 1.29–3.35), perceived fatigue (OR = 1.93; 95% CI 1.21-3.10), recurring falls (OR = 2.01; 95% CI 1.11-3.62) and excessive drowsiness (OR = 1.91; 95% CI 1.11–3.29). The discrimination of the final model was AUC = 0.673 (95% CI 0.619-0.727) (p< 0.001).

**Conclusions:**

The prevalence of dizziness in community-dwelling elderly adults is substantial. It is associated with other common geriatric conditions usually neglected in elderly adults, such as fatigue and drowsiness, supporting its possible multifactorial manifestation. Our findings demonstrate the need to expand the design in future studies, aiming to estimate risk and identify possible causal relations.

## Background

Dizziness is a common disabling symptom among elderly adults [1,2]. It is defined as a subjective feeling of the illusion of movement, a disorientation of the body in space or postural instability [3,4]. Some population-based studies have reported that the prevalence of dizziness in elderly people ranges from 11% to 39% and significantly increases with age [1,3,5]. Data from the National Health and Nutrition examination Survey Study (NHAES) with US adults aged 40 years and older showed that the prevalence of vestibular dysfunction was 49.4%, 68.7% and 84.8% at ages 60–69, 70–79 and 80 and over, respectively [6].

Clinical and social conditions associated with dizziness are heterogeneous, making the identification of underlying causes sometimes ineffective [4,7,8]. In about 40% of dizzy patients after one year of follow-up, family doctors were still unable to specify a diagnosis, treating the complaint of dizziness or vertigo as a diagnosis [9].

Dizziness is a strong predictor of falls, recurring falls [6,10] and disability in elderly adults [1,2]. Several health, psychological and social characteristics were observed in association with a report of dizziness in elderly adults living in the community, such as age [3,6], female gender [3,5,6], low educational level [6], anxiety [11] and depression or depressive symptoms [3,5,11], past myocardial infarction, postural hypotension [11] and cardiovascular disease [3], diabetes [6], poor hearing and vision [5,11], using a large number of medications [3,11], having three or more diseases [3], poor self-health rate [3] and balance and gait disorders [3,11]. Among patients that visited their family physicians due to dizziness it was found that living alone, having little education and presenting cardiovascular disease and hypertension increased the chances of reporting dizziness [9].

Due to its unspecific and complex manifestation in elderly people, dizziness has been considered either a geriatric syndrome [11-13] or a geriatric condition [14], both of them characterized as a multifactorial health condition not related to a specific disease. Despite its similarity, a geriatric syndrome distinguishes from a geriatric condition in its conceptual framework. If dizziness is to be considered a geriatric syndrome it is expected to be age-dependent, to have multiple risk factors and might be correlated with other syndromes, such as falls, frailty and disability. Health care practitioners are faced with the challenge of looking for its underlying causes and, mainly, identifying treatable dizziness-related conditions. A profile of dizziness in elderly adults is still uncertain [15]. We conducted this study to explore the interrelations between dizziness and a broad range of health and geriatric conditions, diseases, geriatric syndromes and demographic characteristics in a representative sample of community-dwelling elderly people.

## Methods

### Study design

Data were obtained using a cross-sectional population-based study based on the FIBRA Network Study (Frailty in Brazilian Older Adults).

### Setting

Elderly participants living at home in an urban area were enrolled through a process of random cluster sampling of census regions. Data were collected from March, 2009 to April, 2010.

### Participants

Both male and female participants aged 65 or older were included. Exclusion criteria were based upon the methodological recommendations proposed by Ferrucci et al. [16]: 1) Severe cognitive impairment according to the Mini-Mental State Examination (MMSE), adjusted for education level [17], as follows: < 17 points for illiterates, < 20 points for those participants with low educational level (one to four years of education), < 24 points for those with four to eight years of education, and < 26 points for participants with nine or more years of education; 2) inability to walk (either temporarily or permanently); 3) localized loss of strength and aphasia due to severe stroke; 4) Parkinson's disease (either severe or unstable); 5) severe hearing or visual impairment; and 6) terminal illness.

The study protocol was approved by the Research Ethics Committee of the Clínicas Hospital of Ribeirão Preto (protocol number 2033/2007) and all participants provided written informed consents.

### Measures and instruments

#### Dizziness

Dizziness was investigated by self-report, established through the following question: “Have you felt dizzy in the last year?” The elderly adults were asked to consider dizziness as any feeling of spinning, rotation, light and heavy head, floating, fuzziness, giddiness or unbalance.

### Demographic characteristics

The demographic variables were: gender, age (brackets of 65–69, 70–74, 75–79 and 80 years and older), years of education classified as less than one year of education, 1 to 4 years, 5 to 8 years and 9 or more years of education. Living alone was categorized dichotomously. The gross monthly family income from paid work or retirement pension was classified into four groups, where 1 represents the minimum wage salary (0.0-1.0; 1.1-3.0; 3.1-5.0; 5.1 and above).

### Diseases

Diseases were identified by self-report, based on the presence of any of the following diseases, diagnosed by a physician, in the last twelve months: heart disease, hypertension, cerebral vascular accident, diabetes, rheumatic diseases, depression and osteoporosis. Co-morbidity was defined as the presence of two or more diseases [17].

### Health and geriatric conditions assessment

Self-rated health was assessed by self-report and participants were asked to evaluate the general status of their health. The possible answers were very good, good, average, bad and very bad. A three category variable was created. Polypharmacy was defined as the concomitant ingestion of four or more regular medications in the last three months [18]. Memory problems were investigated by the self-reporting of frequent difficulty with remembering recent events in the last 12 months dichotomously. The presence of perceived fatigue was measured using two statements from the *Center for Epidemiologic Studies-Depression Scale (CES-D)*[19] .“I feel that I have to make an effort to perform my normal activities” and “I left many of my interests and activities”. The possible responses were "never/rarely" (less than one day), "rarely" (one or two days), "often" (three or four days), "always" (five to seven days). Those who reported three or more days were considered as suffering fatigue. The degree of sleepiness was assessed by the Epworth Sleepiness Scale, cross-culturally adapted and validated [20]. It is a self-administered questionnaire, which assesses the likelihood of dozing off or falling asleep in eight monotonous daily situations, each with a classification of 0 to 3, so that the total range of points goes from 0 to 24. Higher scores indicate excessive daytime drowsiness. The total score of 10 or more points was used to indicate excessive drowsiness [21].

Depressive symptoms were assessed using the short version of the *Geriatric Depression Scale* (GDS), formed by 15 questions [22]. The screening result was considered positive if the participant scored ≥ 5 points.

Fear of falling was measured by the *Falls Efficacy Scale International* (*FES-I*), cross-culturally adapted and validated [23]. It was evaluated by asking questions regarding the participants concern with the possibility of falling while performing 16 activities, each with respective scores of one to four. The total score can range from 16 (no concern) to 64 (extreme concern). The average score was calculated and the results divided between ≤ 22 and > 22 points [24].

### Disability

Disability was evaluated by the Katz Index that measures the need of help to perform six daily activities (bathing, dressing, toileting, transferring, continence and eating). The number of activities that were carried out with total dependency were summed and dichotomously categorized as follows: none or 1 or more activities.

### Geriatric syndromes

Falls were ascertained by self-report of any event in the last year, according to the following definition: a fall is considered an unintentional and unexpected change of position, which makes the individual stay in a lower level, for example, on a piece of furniture or on the floor. This event is not a result of sudden paralysis, epileptic seizure or extreme external force [25]. The reports were categorized into no falls or one or more falls. Participants were considered as recurring fallers if they had experience two or more falls in the previous year.

Frailty was established according to the Fried et al. phenotype [17]. Elderly participants who scored on 3 or more of these criteria were classified as frail: a) Unintentional loss of weight in the last year, bigger than 4.5 kg or than 5% of the body weight, b) Exhaustion or fatigue, indicated by always or almost always responses on any of two items from the Centre of Epidemiological Studies-Depression scale (CES-D) c) low grip strength, indicated by values below the first quintile of the sample, adjusted for gender and BMI measured by a hand dynamometer (SAEHAN® - SH 5001); d) low self-selected walking speed, indicated by values above the 80th percentile of time required to walk a 4.6 m distance, adjusted by height and gender; e) low energy expenditure, a weighted score of kilocalories expended per week; it was indicated by the values below the first quintile of the sample, adjusted by gender, measured by a short version of the Minnesota Leisure Time Activities Questionnaire (Q-MLTPA) [17,26,27]. The participants were asked if they performed the activity, the average frequency of the activity in the past two weeks and the duration of the activity. The energy expenditure, estimated in kilocalories/minute (kcal/min), per each subject, was calculated using the following equation: energy expenditure (kcal/min) = 0.0175 kcal × kg-1 × min-1 × MET × body weight (kg).

### Statistical analyses

The sample size estimated for cities with less than one million inhabitants was 385 participants. Univariate logistic regression estimated the raw association between dizziness and the outcome variables. A screening criterion of p< 0.05 was used to select independent variables to enter into the multiple analysis. Multivariate logistic regression analysis was conducted to estimate the adjusted odds ratios and build the probability model for dizziness. Items with a p< 0.05 were eliminated one by one in sequence of the *p* value. When an item was eliminated, if the change of any remaining parameter estimate (β coefficient) was bigger than 20%, this item remained in the model as a confounder. Poor self-rated health was found to be a confounder during item elimination. Its relationship with the other variables was tested and we identified that the presence of depressive symptoms was able to explain the association between dizziness and poor self-rated health so it was removed from the final model. The odds ratios with the lower and upper 95% confidence intervals (95% CI) and p-values were reported. The fit of the multiple logistic regression model was evaluated by the Hosmer-Lemeshow goodness-of-fit test. Discrimination (the ability to distinguish those who reported dizziness from those who did not) was quantified using the area under the curve [28] of the receiver, operating characteristic curves (ROC curves) in a 95% CI. The statistical SPSS 17.0 package for Windows was used for data analysis.

## Results

**Figure 1:**
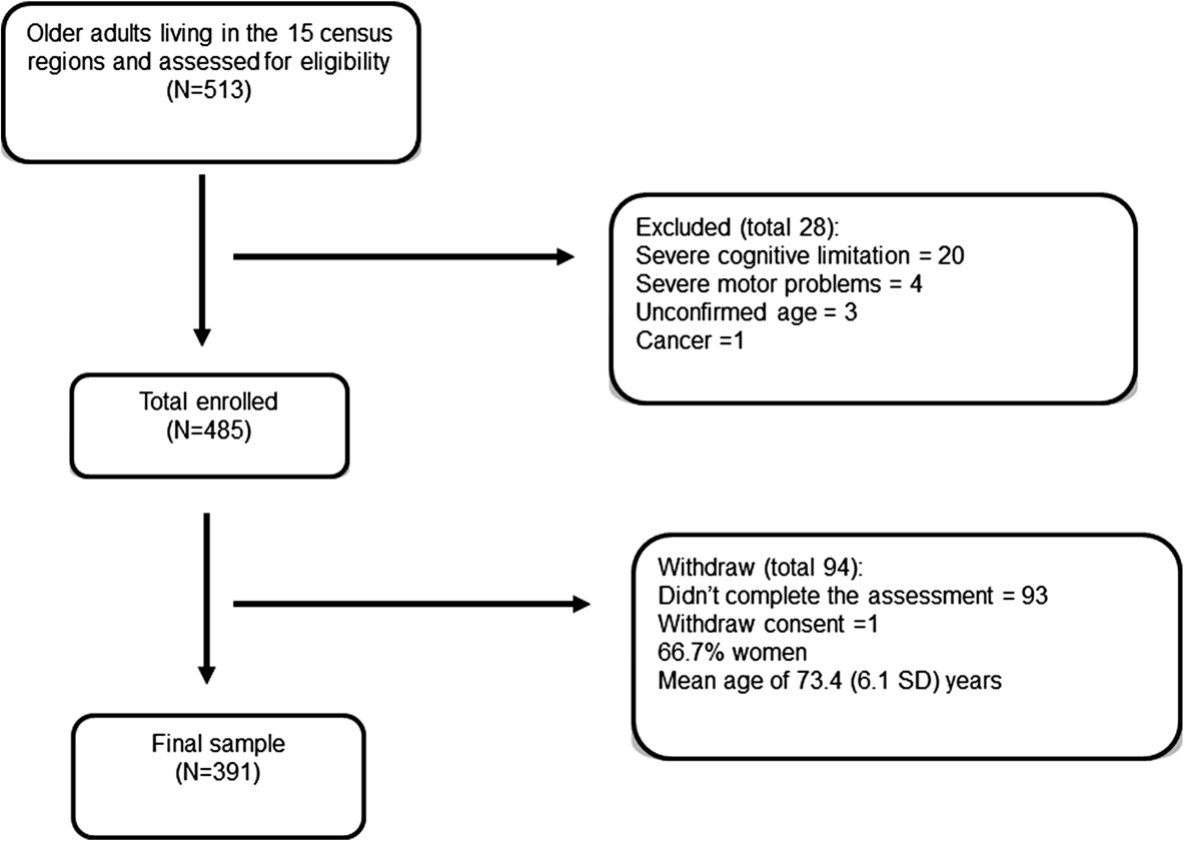
Flowchart of the study sample.

The sample consisted of 391 participants, from which 249 (63.7%) were women, with a mean (standard deviation) age of 72.4 (6.0) years. Overall, 46.1% of the participants considered their health as good or very good. The flowchart of the study sample is shown in Figure 1.

Dizziness during the last year was reported by 45% (n=176) of the elderly people. The average age of the participants who reported dizziness was 72 (5.9) years and among those with dizziness, women represent 71.6% (*p*=0.004). Table 1 provides information on the participants’ characteristics in relation to dizziness and the raw odds ratio derived from the univariate regression analysis. Examining the demographic characteristics no association between dizziness and age, years of education, income level and living alone was observed. Participants with dizziness tend to perceive their health as average, bad and very bad more frequently than those without dizziness. Elderly adults who perceive their health as average were more likely to report dizziness when compared to those who perceive their health as good or very good. Regarding diseases, participants who present heart disease and osteoporosis had significantly higher probability of complaining of dizziness. Among the geriatric conditions perceived fatigue, memory problems, excessive sleepiness, depressive symptoms and fear of falling were significantly associated with dizziness. It was also observed that individuals with falls, recurringt falls, disability and those who exhibited a pre-frail status were more likely to report dizziness in the past year.

**Table 1:** Crude odds ratios for dizziness in 391 community-dwelling older adults

**Correlates**	**No dizzy n (%)**	**Dizzy n (%)**	***p*-Value**	**Odds ratio**	**95% CI**
**Female gender**	123 (57.2)	126 (71.6)	0.003	1.88	1.23-2.88
**Age Group (ref 65–69 years)**					
65-69 years	84 (39.1)	64 (36.4)			
70-74 years	58 (27.0)	60 (34.1)	0.217	1.35	0.83-2.20
75-79 years	41 (19.1)	30 (17.0)	0.890	0.96	0.54-1.70
80 years and over	32 (14.9)	22 (12.5)	0.750	0.90	0.47-1.69
**Years of Schooling (ref Illiterate)**					
Illiterate	52 (24.2)	46 (26.1)			
1 to 4 years	86 (40.0)	77 (43.8)	0.962	1.01	0.61-1.67
5 to 8 years	41 (19.1)	25 (14.2)	0.252	0.68	0.36-1.30
9 years and over	36 (16.7)	28 915.9)	0.690	0.87	0.46-1.65
**Income level (ref up to 1)**^†^					
Up to 1	125 (59.5)	104 (61.9)			
1.0 to 3.0	59 (28.1)	45 (26.8)	0.715	0.91	0.57-1.46
3.1 and over	26 (12.4)	19 (11.3)	0.694	0.87	0.46-1.67
**Living alone (yes)**	28 (13.0)	33 (18.0)	0.122	1.541	0.890–2.668
**Health Perception (ref very good)**					
Very good	117 (54.4)	63 (35.8)			
Average	84 (39.1)	97 (55.1)	<0.001	2.14	1.40-3.27
Bad and Very Bad	14 (6.5)	16 (9.1)	0.059	2.12	0.97-4.63
**Diseases**					
Heart disease	31 (14.4)	39 (22.2)	0.048	1.69	1.04-2.84
Hypertension	145 (67.4)	127 (72.2)	0.314	1.25	0.80-1.93
Stroke	9 (4.2)	8 (4.5)	0.862	1.09	0.41-2.88
Diabetes	46 (21.4)	36 (20.5)	0.820	0.94	0.57-1.54
Arthritis	75 (34.9)	75 (42.6)	0.118	1.38	0.92-2.08
Pulmomary disease	19 (8.8)	12 (6.8)	0.463	0.75	0.35-1.60
Osteoporosis	58 (27.0)	73 (41.5)	0.003	1.91	1.25-2.93
**Geriatric Conditions**					
Fatigue	47 (21.9)	73 (41.5)	<0.001	2.53	1.63-3.93
Polypharmacy	46 (21.4)	41 (23.3)	0.653	1.11	0.69-1.79
Comorbidity	133 (61.9)	121 (68.8)	0.156	1.35	0.89-2.06
Memory problems	90 (41.9)	96 (54.5)	0.013	1.66	1.11-2.49
Excessive sleepiness	30 (14.0)	46 (26.1)	0.003	2.18	1.30-3.64
Fear of falling	129 (60.0)	131 (74.4)	0.003	1.94	1.25-2.99
Depressive symptoms^‡^	44 (20.5)	74 (42.0)	<0.001	2.82	1.80-4.40
**Geriatric Syndromes**					
Falls	71 (33.2)	75 (42.9)	0.050	1.51	0.99-2.28
Recurrent falls	23 (10.7)	41 (23.4)	0.001	2.54	1.45-4.43
No frail (ref)	90 (41.9)	54 (30.7)			
Pre Frail ^§^	101 (47.0)	98 (55.7)	0.031	1.61	1.04-2.50
Frail ^§^	24 (11.2)	24 (13.6)	0.129	1.66	0.83-3.21
Disability (ref 0 ADLs)	16 (7.4)	24 (13.6)	0.047	1.96	1.00-3.82

Notes: MWS= Minimum Wage Salary; ADL = Activities of Daily Living.

^†^ Income Level is presented in Minimum Wage Salaries.

^‡^ Depressive symptoms identified by ≥ 5 points in Geriatric Depression Scale.

^§^ Pre –Frail and frail identified by 1 or 2 and 3 or more of the following criteria, respectively: unintentional weight loss, weaknesses, low physical activity, slow walking speed and fatigue.

^††^ Number of missing values: Income Level = 13 (3.3%), Fall = 2 (0.5%); Recurrent Falls = 2 (0.5%).

In the multivariate adjusted model (Table 2) dizziness was independently associated with depressive symptoms (OR = 2.08; 95% CI: 1.29–3.35), perceived fatigue (OR = 1.93; 95% CI: 1.21-3.10), recurring falls (OR = 2.01; 95% CI: 1.11-3.62) and excessive sleepiness (OR = 1.91; 95% CI: 1.11–3.29) (Hosmer and Lemeshow test = 0.999 and Nagelkerke R square test = 0.137). The discrimination of the final model was AUC = 0.673 (95% CI: 0.619-0.727) (p< 0.001).

**Table 2:** Multivariate model for dizziness, in 391 community-dwelling older adults

**Variables**	**β coefficient**	**Odds ratio**	**95% CI**	***p*-value**
Depression†	0.733	2.08	1.29-3.35	0.003
Fatigue	0.662	1.93	1.21 – 3.10	0.006
Sleepiness†	0.649	1.91	1.11 – 3.29	0.019
Recurrent falls	0.699	2.01	1.11 -3.62	0.020

†Depression was identified by ≥5 points in the Geriatric Depression Scale and Sleepiness was identified by ≥ 10 points in the Epworth Sleepiness Scale.

^‡^Number of missing values: Recurrent Falls = 2 (0.5%).

Hosmer and Lemeshow test = 0.999; Nagelkerke R square = 0.137.

Area under the curve (AUC) = 0.673 (95% CI, 0.619-0.727).

## Discussion

This study can be considered one of the first ones to investigate dizziness in a representative sample of elderly adults living in the community in a developing country. We observed a higher prevalence of dizziness when compared with other population-based studies [4,5,11]. It is suspected that this prevalence difference is due to the heterogeneity on the scope of the questions used to identify participants with dizziness in population-based studies [3,29]. Elderly adults with depressive symptoms, perceived fatigue, excessive sleepiness and recurring falls were more likely to report dizziness in the past year. Among the outcomes in the final model, the presence of five or more depressive symptoms was the main contributor for reporting dizziness, followed by falls and fatigue. Our findings suggest that dizziness in community-dwelling elderly adults might be considered a complex geriatric condition, rather than a geriatric syndrome which co-occurs and interacts with other common conditions in elderly adults, exposing them to adverse health events, such as recurring falls.

Unexpectedly, we observed a lack of association between advancing age and dizziness. However, other important studies have come across with this same outcome [5,11,13]. We noticed a slight but not significant increase in the prevalence of dizziness among the young old, but after the age of 75 the occurrence of dizziness decreased and this trend was mainly detected for women. Also in our sample the oldest participants were quite healthy. Only 5.6% of then reported to have a poor or very poor health, which could in part explain this lack of association. It also has been discussed that dizziness might be age-concomitant rather than age-dependent [30].

Curiously, dizziness interacted with fatigue, sleepiness and depressive symptoms that are clinically heterogeneous geriatric conditions, which are likely to share some etiological factors and pathological pathways [11-13]. Fatigue can be considered a symptomatic manifestation of several subclinical diseases and is related, in the elderly, to an increased state of chronic inflammation, physiological dysregulation or an increased workload to maintain homeostasis [31], suggesting that it represents a general state of physiological change, and, ultimately, may be a marker of declined functional reserve in the aging [32] .It has been reported that fatigue can be a secondary problem among patients with vestibular pathology, together with muscular pain and increased muscle tension and chronic anxiety [33]. Hardy and Studenski studied five qualities of fatigue among older adults with chronic conditions and identified that a substantial prevalence of participants (70%) reported any type of fatigue. Additionally, the majority of participants complaining of fatigue reported multiple qualities (emotional, cognitive, sleepiness, low energy, weakness and tiredness) despite the present chronic disease [34]. We suggest that dizziness and fatigue co-occur and may be expressed in different combinations of symptoms, becoming complex geriatric conditions with a common underlying process, such as increased inflammation or disordered homeostasis [34] rather than a discrete condition.

Regarding, the association between excessive sleepiness and dizziness the rationale is much more unclear. Literature is relatively uncertain about the true clinical meaning of excessive sleepiness among the elderly. It is often seen as a non-specific complaint and closely related to depression [35]. In a prospective cohort study excessive day time sleepiness predicted the incidence of depression, appearing as a vulnerability factor for acute and chronic depressive episodes. However, a recent, case controlled study which followed patients in tertiary outpatient otoneurology clinic found that there was a significant association between idiopathic dizziness, excessive sleepiness and sleep apnoea. Nearly 39% of the patients with excessive sleepiness, identified through the *Epworth Sleepiness Scale* (>10 points), had idiopathic dizziness compared to 14.7% in the control group [36]. In another study in which the impact of excessive daytime sleepiness on the functionality of elderly persons was analyzed the authors suggested that disability resulting from sleepiness was not primarily linked to depression or dementia, as these elderly persons were carefully excluded from the sample, together with those with any other clinical conditions that could possibly hamper functionality [37]. There seems to be an independent relation between dizziness, drowsiness and fatigue, however the aetiology of these relations remains obscure. It is worth noticing that the use of specific medications may have some effect on the association between dizziness, fatigue and drowsiness. In a recent cross-sectional study it was identified that dizziness and fatigue were among the clinical complaints or reasons for elderly adults’ hospitalization, possibly associated with inappropriate use of medications [38]. However, in a large epidemiology study in Sweden, the use of number of medications in addition to the use of specific medications and diseases was associated with dizziness and faintness [29].

It was observed that depression measured by the presence of depressive symptoms was strongly associated with dizziness in our study and was the main contributing factor in our final model. There is a well-known association between vestibular dysfunction and depression [3,5,11,39]. Elderly people with more depressive symptoms are more likely to have dizziness when compared to those with fewer symptoms [3,11] Elderly people with dizziness tend to have some consequences such as decreased functional performance in daily activities, anxiety and insecurity, which can, in time, change their mood, increasing the chances of depression. Additionally, the relationship between dizziness and depression is complex and can also be partly explained by the adverse effect of antidepressant medications.

We observed that participants with dizziness were twice as likely to report recurring falls in the previous year compared with those who had a single fall or no fall at all. Dizziness was identified as a strong predictor of recurring falls in the elderly [10,40,41] and its underlying mechanism is likely to be a consequence of a cumulative decrease in the physiological systems related to balance and postural control, mainly in the vestibular system, which, ultimately, can be affected by age itself, as well as by a variety of vestibular disorders. It has been suggested that the effect of age on postural instability may be, in part, mediated by vestibular dysfunction [6].

Pre-frail and non-frail elderly adults were more likely to report dizziness, but this association was no longer observed when other outcomes entered in the final model. Curiously, the association with dizziness was not observed in the frail participants, supposedly the most vulnerable group. Recently, Dros et al. [15] conducted an analysis of the main components to establish a classification of diagnostic profiles of dizziness based on empirical data and identified six components and frailty accounted for the biggest total variance, followed by the psychological and cardiovascular ones. However the authors highlighted that most patients were classified in more than one profile, signalizing the co-occurrence of many dizziness related-factors. We used the Fried phenotype, which is based on large physical components and is not expected to cover all possible manifestations of frailty in elderly adults [42]. Some studies have shown a strong evidence that disability is independently associated with dizziness [1,2] but we did not observe this in our study. We suspect that the instrument that we used was not capable of capturing those elderly adults with a less severe disability status, since the Katz instrument measures the need for assistance to perform basic daily activities. Aggarwal et al. [1] have also found a higher association between dizziness and disability in the Rosow-Breslau and Nagi measures, but not in the Katz ADL Scale.

As for disease, we did not observe any association between hypertension or cardiovascular disease and dizziness. This association has been more commonly identified among patients seeking medical assistance [9,13] than in elderly adults living in the community [6].

We tried to identify if some outcomes of health inequality were associated with dizziness, since this is one of the first studies to explore this health condition in a developing country; however, no significant differences in social-demographic variables were identified between those who reported dizziness and those who did not. There is some discrepancy in the literature concerning the association between dizziness and demographic outcomes. Some studies have identified the association between age and gender[1,3,9] with dizziness, but not others [2,5] and a relationship between dizziness and the educational level [6] has also been observed, but none with wealth [5].

Our study has a cross-sectional design limitation, preventing the establishment of relationships between causality and estimation of risk and we did not investigate the type of medications the participants were taking profoundly, either. Furthermore, there could possibly be a memory bias, since participants were asked to report dizziness at any time in the previous year. The identification of fatigue based on questions from an instrument developed to identify depression may also have increased the chances of association with dizziness. Nevertheless, these two questions of the CED-S have been systematically used in the literature to measure fatigue/exhaustion in elderly adults [43,44]. The strength of this study lies on the fact that it was a population-based study that investigated the complaint of dizziness in a representative sample of community-dwelling elderly persons using a comprehensive range of outcomes and explored the several symptoms related to dizziness.

Due to its interaction with other complex complaints and health conditions we suggest that dizziness in elderly adults is a multifactorial geriatric health condition and health care practitioners should conduct a comprehensive assessment in other to identify its associated conditions, together with strategies to investigate the underlying cause-related specific diseases.

## Conclusion

This study shows that the prevalence of dizziness is substantial. Dizziness is associated with conditions usually neglected in the elderly, such as fatigue and drowsiness, which are known to hamper the functional capacity and quality of life in the long term. Our findings demonstrate the need to expand the design in future studies, aiming to estimate risk and identify possible causal relationships.

## Competing interests

The authors declare no conflicts of interest.

## Authors’ contributions

SAM, WJSS, EF and MRP were responsible for data acquisition and data analysis and interpretation, drafting the article and final approval of the manuscript. MRP and EF were local coordinators of the multicentre study and also responsible for general supervision of the research group. EF was one of the coordinators for the FIBRA Study nationally and responsible for the acquisition of funding. All authors read and approved the final manuscript.

## Pre-publication history

The pre-publication history for this paper can be accessed here:

http://www.biomedcentral.com/1471-2318/13/4/prepub
